# Design and base-line evaluation of financial incentives to increase effective coverage of priority health interventions funded by Seguro Popular, Mexico

**DOI:** 10.1186/1472-6963-14-S2-P48

**Published:** 2014-07-07

**Authors:** Miguel-Angel Gonzalez-Block

**Affiliations:** 1 Director de Diseño de Politicas y Programas de Salud, PwC, Distrito Federal, Mexico

## Purpose

To present a ground-breaking results-based financial incentives experiment.

## Content

Mexico’s Seguro Popular has contributed towards universal financial risk protection. However, coverage is low for chronic diseases, with only 26% and 30% of affiliate adult men and women, respectively, having access to preventive care. The state of Hidalgo’s Seguro Popular affiliating 3.4 million people introduced a results-based financial incentive scheme to improve performance of key outputs and outcomes. A total of 25 indicators were chosen: 20 for PHC and 5 for hospital care, covering diabetes, cardiovascular health, prenatal care, breast cancer screening, oral health, family planning, chronic disease prevention and interpersonal quality.

Indicator base-line was set using survey data; caps were defined using an expert panel to identify provider control over resources and outcomes. A standardized point system was devised to compare providers and to facilitate monitoring, using health gain, costs and expert priorities. The cash value per point was set for each provider based on the size and socioeconomic level of their coverage target. To encourage coverage of the most difficult to reach, performance below a threshold -set higher for hospitals- leads to discounts, while performance at each of four superior levels increases point value geometrically. The size of the incentive fund was estimated at 10% of the payer’s financial capacity. The impact of incentives on increased activity and health care costs was estimated. A case-control evaluation base-line was established.

## Significance

Success with the scheme promises to speed-up the introduction of financial incentive schemes for Seguro Popular in other states in Mexico and to provide much needed experience for other health systems around the world.

## Target audience

This presentation will be of interest to decision makers, consultants and researchers involved in the design of results-based financing innovations as a strategy to increase effective coverage of high-priority health interventions.

